# Effect of Low Diastolic Blood Pressure to Cardiovascular Risk in Patients With Ischemic Stroke or Transient Ischemic Attacks Under Different Systolic Blood Pressure Levels

**DOI:** 10.3389/fneur.2020.00356

**Published:** 2020-05-27

**Authors:** Zimo Chen, Jinglin Mo, Jie Xu, Liye Dai, Aichun Cheng, Gulbahram Yalkun, Anxin Wang, Xia Meng, Hao Li, Yongjun Wang

**Affiliations:** ^1^Department of Neurology, Beijing Tiantan Hospital, Capital Medical University, Beijing, China; ^2^China National Clinical Research Center for Neurological Diseases, Beijing, China; ^3^Center of Stroke, Beijing Institute for Brain Disorders, Beijing, China; ^4^Beijing Key Laboratory of Translational Medicine for Cerebrovascular Disease, Beijing, China

**Keywords:** diastolic blood pressure, ischemic stroke, secondary prevention, blood pressure monitoring, composite events

## Abstract

**Background:** In the context of recently updated strategies of pressure management, there is a paucity of evidence on the effect of diastolic blood pressure (DBP) level on adverse events among stroke patients. This study aimed to examine the effect of low DBP (<60 mmHg) under different levels of systolic blood pressure (SBP) on the risk of composite events and stroke recurrence among patients with ischemic stroke (IS) or transient ischemic attack (TIA).

**Material and Methods:** This study was conducted in 2,325 patients with IS or TIA. DBP values were categorized into <60, 60–70, 70–80 (reference), 80–90, and ≥90 mmHg in the main sample and were further categorized as <60 and ≥60 mmHg (reference) when patients were stratified according to SBP levels (<140, <130, and <120 mmHg). The outcomes were defined as recurrent stroke and cumulative composite events (defined as the combination of nonfatal myocardial infarction, nonfatal congestive heart failure, and death) at 1 year.

**Results:** During 1 year of follow-up, a total of 95 composite events and 138 stroke recurrences were identified. The patients with low DBP showed a significantly higher risk of composite events [hazard ratio (HR) = 4.86, 95% confidence interval (CI) = 2.54–8.52], especially the elderly patients (≥60 years); however, this result was not observed for stroke recurrence (HR = 0.90, 95% CI = 0.46–1.74). With the reduction of the SBP levels, the proportion of patients with low DBP increased (6.87, 12.67, and 34.46%), and the risk for composite events persisted.

**Conclusions:** Along with the new target levels of SBP suggested by updated criteria, there is a trend for DBP to be reduced to a harmfully low level, which was associated with an increased risk of composite events among patients with IS or TIA.

## Introduction

Stroke is a leading cause of mortality and disability worldwide, considerably contributing to social and economic burden, and hypertension has been widely recognized as one of the most powerful risk factors of stroke (1–4). Blood pressure (BP) lowering has been considered an important measure for preventing stroke and other cardiovascular diseases (5–8). The landmark Systolic Blood Pressure Intervention Trial (SPRINT) has demonstrated that compared with <140 mmHg, intensively lowering systolic BP (SBP) to <120 mmHg resulted in lower rates of major cardiovascular events (9). Thereafter, the 2017 American College of Cardiology/American Heart Association (ACC/AHA) guidelines redefined hypertension to adopt a lower BP criterion, from 140/90 mmHg previously to 130/80 mmHg (8). Thus, it is reasonable to question whether these strategies for BP management are also appropriate for patients with stroke (10).

Previous studies mainly focused on the harms of SBP, resulting in more concern of SBP than diastolic BP (DBP) for risk assessment and treatment (11, 12). Further, the Framingham Heart Study and other studies have demonstrated a more important role of SBP than DBP as an independent risk predictor for coronary events and stroke (13–15). However, in one recent study, Flint et al. reported that the burden of diastolic hypertension can also independently predict the risk of adverse cardiovascular events, thus calling for the concern of DBP level (16). Notably, owing to the known J-curve between DBP and cardiovascular events, the harms of low DBP should not be ignored (17). It is also worth noting that intensive SBP reduction will inevitably lower the DBP, which was supported by a secondary analysis of SPRINT among elderly participants, showing that the intensive-therapy group exhibited a reduction of DBP from a mean of 71.5 mmHg at baseline to 62 mmHg during active antihypertensive treatment (18). There is, however, a paucity of evidence on the correlation between DBP level and the risk of adverse events in patients with stroke, especially under the condition of controlled SBP as recommended by the above strategies (19).

The current study aimed to examine the effect of low DBP (defined as <60 mmHg in this study) (20, 21) under different levels of SBP (<140, <130, and <120 mmHg) on the cardiovascular risk among patients with ischemic stroke (IS) or transient ischemic attack (TIA).

## Materials and Methods

### Study Cohort and Participants

The Blood Pressure and Clinical Outcome in Stroke Survivors (BOSS) study is a nationwide, hospital-based, longitudinal cohort study conducted in 61 hospitals in China. The details of the study design, rationale, and baseline characteristics have been previously described (22). Briefly, 2,608 patients (aged 18 years or older) with IS or TIA within 7 days of the index event were enrolled between October 2012 and February 2014 (baseline) for BP monitoring and followed up for 1 year for clinical outcomes.

The study was approved by the central institutional review board of Beijing Tiantan Hospital, as well as ethical committees at the 61 participating hospitals, in compliance with the Declaration of Helsinki. All patients or the designated relatives signed the written informed consent form.

### Blood Pressure Assessment and Classification

After admission, each enrolled patient was provided a semiautomatic upper-arm BP monitor (HEM-4030; Omron, Kyoto, Japan). Nurses trained the patients or their accompanying relatives on the use of the device. During hospitalization, BP was measured twice daily by the patients themselves or their relatives, according to the standard measurement method recommended by the American Heart Association (23). BP data were recorded in an assigned hospitalization BP diary. At discharge, the patients continued to measure their BP twice daily at home, using the assigned Omron BP monitor from the first day after discharge to 3 months after admission, with the assigned home BP diary. The recorded data from hospitalization BP diary and home BP diary of 3 months after admission were then used to calculate the averaged 3-month BP value for analysis.

In the overall population, DBP values were categorized into 10-mmHg increments: <60, 60–70, 70–80, 80–90, and ≥90 mmHg. We chose the category of 70–80 mmHg as the reference. When patients were further stratified according to SBP categories (<140, <130, and <120 mmHg), the DBP values were categorized as <60 and ≥60 mmHg, with ≥60 mmHg as the reference.

### Data Collection

Other baseline information, including age, sex, smoking, alcohol drinking, medical history, and medications, was obtained through a nurse-administered standardized questionnaire at the time of admission. History of stroke was defined as previous IS, intracerebral hemorrhage, and subarachnoid hemorrhage confirmed by medical records. History of coronary heart disease was defined as a reported history of myocardial infarction or cardiac surgery or a final diagnosis of myocardial infarction at discharge. History of hypertension was defined as previous hypertension and use of antihypertensive medication. History of diabetes mellitus was defined as self-reported physician-diagnosed diabetes mellitus or use of antidiabetic drugs. History of dyslipidemia was defined as self-reported physician-diagnosed dyslipidemia or use of lipid-lowering agents.

### Assessment of Clinical Outcomes

The patients were followed up at 3 months through a face-to-face interview and at 12 months via a telephone survey. Patients with nonfatal events were asked to return for a face-to-face follow-up or were visited at home. The clinical outcomes were defined as cumulative composite events and recurrent stroke (ischemic or hemorrhagic) at 1 year.

In the current study, composite events were defined as the combination of nonfatal myocardial infarction, nonfatal congestive heart failure, and death of any cause. Death certificates were obtained for deceased participants, and hospital data were abstracted for all vascular events.

Stroke recurrence was defined as a new stroke event or rapid worsening of an existing focal neurologic deficit lasting >24 h [an increase in the National Institutes of Health Stroke Scale (NIHSS) score by ≥4 points compared with the baseline NIHSS score], accompanied by new ischemic changes on magnetic resonance imaging or computed tomography of brain (24).

### Statistical Analysis

Categorical variables were expressed as absolute or relative frequencies. Continuous variables are presented as means ± standard deviations. Multivariable Cox regression analyses were performed to evaluate the relationship between the DBP category and the outcomes and to calculate the multivariable-adjusted hazard ratios (HRs) and 95% confidence intervals (CIs) for clinical outcomes. The adjustment variables included age, sex, medical history (hypertension, diabetes mellitus, hyperlipidemia, and coronary heart disease), and medication at discharge (antiplatelet, statin, and antihypertension therapies).

All analyses were performed with SAS 9.4 (SAS Institute, Cary, NC, USA). Two-sided *p*-values of 0.05 were considered statistically significant.

## Results

**Figure 1:**
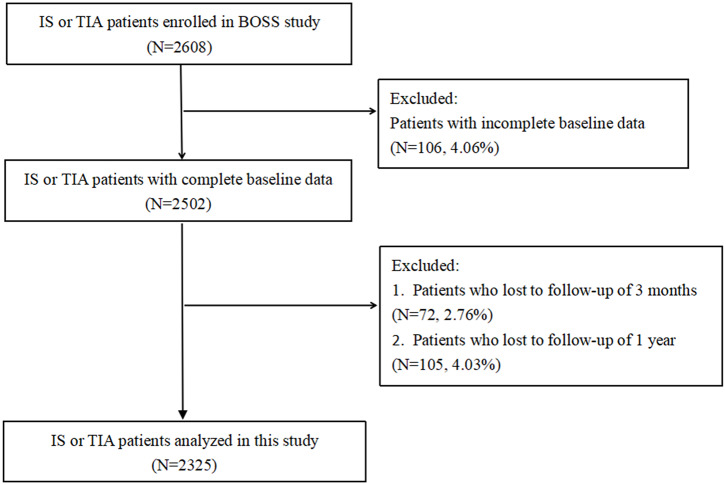
Study flowchart. BOSS, Blood Pressure and Clinical Outcome in Stroke Survivors; IS, ischemic stroke; TIA, transient ischemic attack.

Among the 2,608 patients recruited from the BOSS study, 106 (4.06%) patients with incomplete baseline data and 72 (2.76%) and 105 (4.03%) patients who were lost to follow-up at 3 months and 1 year, respectively, were excluded. Finally, a total of 2,325 patients were successfully enrolled for the final analysis. The flowchart of patient selection is presented in Figure 1. The average age was 62.52 ± 11.06 years (males, 67.23%).

Table 1 summarizes the baseline characteristics of the six subgroups, defined by DBP categories (<60, 60–70, 70–80, 80–90, and ≥90 mmHg). Generally, patients in the lower DBP category were older and more likely to be women. In addition, they were more likely to have a history of diabetes mellitus and less likely to be smokers, to be alcohol drinkers, and to have a history of hypertension.

**Table 1:** Characteristics of patients at baseline by DBP categories.

**Variable**	**DBP <60 mmHg**	**DBP 60–70 mmHg**	**DBP 70–80 mmHg**	**DBP 80–90 mmHg**	**DBP ≥90 mmHg**	***p*-value**
	***n* = 119**	***n* = 219**	***n* = 1,026**	***n* = 764**	***n* = 197**	
Mean ± SD	57.5 (2.1)	66.5 (2.6)	75.4 (2.7)	84.0 (2.8)	99.1 (12.3)	
Age, years	65.62 ± 10.65	66.91 ± 10.82	63.80 ± 10.79	60.33 ± 10.66	57.57 ± 11.02	<0.001
Female, *n* (%)	43 (36.13%)	95 (43.38%)	344 (33.53%)	221 (28.93%)	59 (29.95%)	0.001
Smoker, *n* (%)	37 (31.09%)	60 (27.4%)	307 (29.92%)	290 (37.96%)	76 (38.58%)	0.001
Drinker, *n* (%)	46 (38.66%)	64 (29.22%)	360 (35.09%)	327 (42.80%)	90 (45.69%)	<0.001
NIHSS, median (IQR)	3 (1, 5)	2 (0, 4)	2 (1, 4)	2 (1, 4)	2 (1, 5)	0.307
IS, *n* (%)	107 (89.92%)	187 (85.39%)	936 (91.32%)	675 (88.35%)	175 (89.29%)	0.070
SBP, mmHg	125.86 ± 10.44	128.91 ± 12.64	131.83 ± 9.85	138.16 ± 9.79	135.69 ± 22.91	<0.001
**MEDICAL HISTORY**, ***N*** **(%)**						
Stroke	35 (29.41%)	53 (24.20%)	230 (22.42%)	181 (23.69%)	64 (32.49%)	0.026
Hypertension	75 (63.03%)	140 (63.93%)	680 (66.28%)	581 (76.05%)	160 (81.22%)	<0.001
Diabetes mellitus	35 (29.41%)	51 (23.29%)	231 (22.51%)	139 (18.19%)	46 (23.35%)	0.029
Dyslipidemia	15 (12.61%)	30 (13.70%)	103 (10.04%)	67 (8.77%)	27 (13.71%)	0.099
Coronary heart disease	11 (9.24%)	28 (12.79%)	114 (11.11%)	80 (10.47%)	31 (15.74%)	0.250
Congestive heart failure	0 (0)	2 (0.91%)	9 (0.88%)	5 (0.65%)	0 (0)	0.575
**HISTORY OF MEDICATIONS**, ***N*** **(%)**						
Antiplatelet therapy	33 (27.73%)	51 (23.29%)	203 (19.79%)	161 (21.07%)	45 (22.84%)	0.271
Statin therapy	16 (13.45%)	25 (11.42%)	113 (11.01%)	75 (9.82%)	24 (12.18%)	0.713
Antihypertensive therapy	56 (47.06%)	111 (50.68%)	521 (50.78%)	440 (57.59%)	131 (66.50%)	<0.001
Anticoagulant therapy	2 (1.68)	0 (0)	6 (0.58)	3 (0.39)	2 (1.02)	0.282
**MEDICATIONS AT DISCHARGE**, ***N*** **(%)**						
Antiplatelet therapy	104 (87.39%)	200 (91.32%)	970 (94.54%)	724 (74.76%)	178 (90.36%)	0.003
Statin therapy	96 (80.97%)	185 (84.47%)	894 (87.13%)	635 (83.12%)	152 (77.16%)	0.003
Antihypertensive therapy	65 (54.62%)	136 (62.10%)	670 (65.3%)	534 (69.9%)	143 (72.59%)	0.002

*SBP, systolic blood pressure; DBP, diastolic blood pressure; IS, ischemic stroke; NIHSS, National Institutes of Health Stroke Scale; IQR, interquartile range*.

During 1 year of follow-up, a total of 95 (4.09%) patients with composite events and 138 (5.94%) patients with stroke recurrence were identified. Table 2 shows that after multivariable adjustment, compared with patients in the DBP 70–80 mmHg category, the subgroup with DBP <60 mmHg showed a significantly higher risk of composite events (HR = 4.86, 95% CI = 2.54–8.52); however, this result was not observed for stroke recurrence (HR = 0.90, 95% CI = 0.46–1.74). When the patients were further stratified by age, the correlation between low DBP and risk for composite events remained in the subgroup of older patients (age ≥60 years), but not in patients aged <60 years. For the outcome of stroke recurrence, patients <60 years showed a significantly higher risk of stroke recurrence when the DBP was 80–90 and ≥90 mmHg.

**Table 2:** Hazard ratios and 95% confidence intervals of composite events and stroke recurrence by DBP categories.

	**Composite events**				**Stroke Recurrence**			
	**Cases**	**Event rate**	**Unadjusted HR (95% CI)**	**Adjusted HR (95% CI)**	**Cases**	**Event rate**	**Unadjusted HR (95% CI)**	**Adjusted HR (95% CI)**
**TOTAL COHORT**								
DBP <60 mmHg	19/119	15.97%	4.86 (2.67–8.83)	4.65 (2.51–8.52)	11/119	9.24%	1.03 (0.54–1.98)	0.90 (0.46–1.74)
DBP 60–70 mmHg	10/219	4.57%	1.68 (0.82–3.47)	1.40 (0.67–2.91)	8/219	3.65%	0.60 (0.29–1.26)	0.53 (0.25–1.11)
DBP 70–80 mmHg (reference)	28/1,026	2.73%	1	1	61/1,026	5.95%	1	1
DBP 80–90 mmHg	29/764	3.80%	1.32 (0.79–2.23)	1.57 (0.92–2.67)	45/764	5.89%	0.93 (0.63–1.37)	1.03 (0.69–1.52)
DBP ≥90 mmHg	9/197	4.57%	1.64 (0.77–3.47)	1.91 (0.88–4.42)	13/197	6.60%	1.04 (0.57–1.89)	1.15 (0.62–2.12)
**AGE** ** <60 YEARS**								
DBP <60 mmHg	3/28	10.71%	5.95 (1.48–23.96)	3.63 (0.80–16.41)	2/28	7.14%	2.26 (0.47–10.78)	1.91 (0.39–9.26)
DBP 60–70 mmHg	3/57	5.26%	3.31 (0.83–13.25)	2.58 (0.62–10.63)	2/57	3.51%	1.54 (0.33–7.26)	1.28 (0.27–6.10)
DBP 70–80 mmHg (reference)	6/362	1.66%	1	1	8/362	2.21%	1	1
DBP 80–90 mmHg	10/370	2.70%	1.60 (0.58–4.39)	1.34 (0.48–3.81)	19/370	5.14%	2.32 (1.01–5.31)	2.31 (1.00–5.34)
DBP ≥ 90 mmHg	3/111	2.70%	1.60 (0.40–6.41)	1.07 (0.26–4.39)	8/111	7.21%	3.17 (1.19–5.45)	2.97 (1.10–8.05)
**AGE** **≥** **60 YEARS**								
DBP <60 mmHg	16/91	17.58%	4.29 (2.21–8.33)	4.80 (2.44–9.42)	9/91	9.89%	0.78 (0.37–1.61)	0.66 (0.26–1.67)
DBP 60–70 mmHg	7/162	4.32%	1.29 (0.55–3.03)	1.30 (0.55–3.07)	6/162	3.70%	0.45 (0.19–1.04)	0.74 (0.46–1.19)
DBP 70–80 mmHg (reference)	22/664	3.31%	1	1	53/664	7.98%	1	1
DBP 80–90 mmHg	19/394	4.82%	1.37 (0.74–2.53)	1.31 (0.70–2.45)	26/394	6.60%	0.75 (0.47–1.20)	0.45 (0.19–1.06)
DBP ≥90 mmHg	6/86	6.98%	2.04 (0.82–5.03)	1.72 (0.68–4.34)	5/86	5.81%	0.67 (0.27–1.68)	0.66 (0.26–1.67)

*Adjusted for age, sex, medical history (hypertension, diabetes mellitus, dyslipidemia, and coronary heart disease), and medication at discharge (antiplatelet, antilipid, and antihypertension)*.

*DBP, diastolic blood pressure; HR, hazard ratio; CI, confidence interval*.

**Figure 2:**
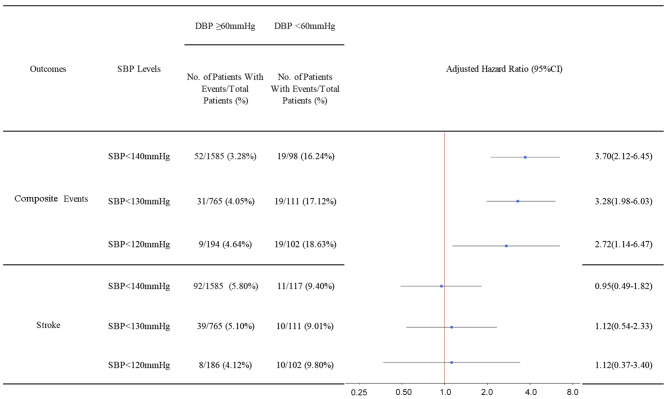
Hazard ratios and 95% confidence intervals of composite events and stroke recurrence according to low DBP under different SBP levels. Adjusted for age, sex, medical history (hypertension, diabetes mellitus, dyslipidemia, and coronary heart disease), and medication at discharge (antiplatelet, antilipid, and antihypertension). SBP, systolic blood pressure; DBP, diastolic blood pressure.

Figure 2 presents the changes in the proportion of patients with low DBP with the reduction of SBP levels (<140, <130, and <120 mmHg). It was observed that the proportions of patients with DBP <60 mmHg increased as the SBP decreased (6.87, 12.67, and 34.46%).

**Figure 3:**
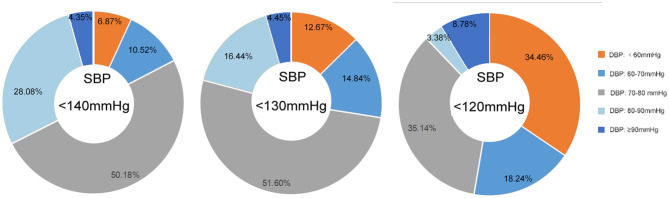
Proportion of patients with different DBP levels under different SBP levels (<140, <130, and <120 mmHg). SBP, systolic blood pressure; DBP, diastolic blood pressure; CI, confidence interval.

Figure 3 shows the analyses of patients stratified by SBP levels (<140, <130, and <120 mmHg). Compared with the subgroup with DBP ≥60 mmHg, the subgroup with DBP <60 mmHg showed a significantly higher risk for composite events in all SBP categories. Nevertheless, an increased risk of stroke recurrence was not observed among patients with low DBP.

Supplemental Figures 1, 2 show the results of analyses after excluding adverse events occurring within the first 3 months, namely, the period of pressure monitoring. Owing to the low event rate and lack of statistical power, only the event rates were presented. It was still observed that patients with low DBP had a higher rate of cardiovascular events, not only in the total population but also under different SBP levels.

## Discussion

In this study, we demonstrated that, compared with the medium level of DBP (70–80 mmHg), low DBP (<60 mmHg) was associated with a significantly higher risk of composite events, especially among the elderly (the main population affected by isolated systolic hypertension) (25–27). However, we failed to find the same trend for stroke recurrence. Notably, with the reduction of SBP levels (<140, <130, and <120 mmHg), an increasing proportion of patients with low DBP was observed, for whom the negative effects of low DBP on composite events persisted, indicating that achieving the optimal SBP level in patients with IS or TIA may be accompanied by harmfully low DBP.

Although a lower SBP target than that previously recommended (140 mmHg) has been endorsed by SPRINT and the 2017 ACC/AHA guidelines, its benefits have not been widely recognized (9, 10). In the 2018 European Society of Cardiology/European Society of Hypertension guidelines, the target for treating high BP remained unchanged (office BP ≥ 140/90 mmHg) (28). Despite the considerable clinical benefits of antihypertensive therapy, one of the major concerns following aggressive SBP reduction is the potential risk of myocardial damage caused by the accompanying lowered DBP (18, 29). Several previous studies have verified the influence of low DBP on adverse cardiac outcomes (20, 30, 31). The underlying pathophysiological mechanism could be explained by the theory that coronary blood flow primarily occurs during diastole and that heart perfusion might be compromised at low DBP values. In this study, we found that compared with that at the SBP level of 140 mmHg, the proportion of patients with low DBP at the SBP level recommended by the 2017 ACC/AHA guidelines and SPRINT doubled and quintupled, respectively, and the risk of composite events persisted in these patients. On the contrary, we failed to find a significant effect of low DBP on the risk of stroke recurrence. Similarly, previous studies were not able to demonstrate the correlation between low DBP and stroke, despite the J-shaped relationship between DBP and composite events (21, 32). Our findings indicated the importance of paying attention to the increasing proportion of patients with low DBP relative to the risk of composite events when adopting a more intensive SBP-lowering treatment among patients with IS or TIA, especially in the post-SPRINT era.

In the elderly, as the main population affected by stroke, isolated systolic hypertension is the most common hemodynamic form of hypertension (25–27). In our study, patients aged ≥60 years were observed to be the major group experiencing the harms of low DBP. In addition, we also observed an increased risk of stroke recurrence in patients aged <60 years with a higher DBP (80–90 or ≥90 mmHg), probably because of the possibility that DBP and SBP are more likely to show parallel changes in younger patients and because higher DBP often reflects a tendency of higher SBP, an important risk factor for stroke recurrence (33, 34). The results suggested that pressure-lowering therapy should be carefully applied in elderly patients with IS or TIA; however, for younger patients, more aggressive therapy seems likely to reduce the risk of stroke recurrence without the accompanying risk of composite events. In other words, age might serve as an important factor linking DBP level to the cardiovascular risk.

The main strength of this study was that compared with the pressure measurement at a single time point, the averaged BP value over 3 months of monitoring might be a more reliable indicator of long-term BP control. Our blood pressure measurement is also better correlated with cardiovascular events than other clinic measurements. It was a better prognostic indicator for long-term cardiovascular events and mortality and can identify white coat and masked hypertension. It provided better sensitivity and might be suitable for identifying abnormal blood pressure given its relative ease of use and availability compared with other clinic measurements like ambulatory monitoring.

However, our main analysis included the adverse events that occurred within the period of pressure monitoring, which ignored the time sequence of pressure monitoring and adverse events. Therefore, we further performed a sensitivity analysis including only the outcomes from 4 months to 1 year of follow-up. Although lacking statistical power, the event rate showed the same trend as in the main analysis. Besides, because this study included Chinese patients only, it may limit the generalizability of the conclusions to other populations. Additionally, this study was an observational study, and the possibility of residual confounding cannot be fully eliminated, in spite of the multivariable regression analyses for several important potential confounders. Moreover, our study does not have the information about cardiovascular mortality, so we used all-cause mortality instead. Further studies using cardiovascular mortality as the endpoint are needed to verify the findings of this study.

## Conclusions

In summary, our study demonstrated the correlation between the reduction of SBP levels (<140, <130, and <120 mmHg) and increasing proportion of patients with low DBP, indicating that a strict SBP control targeting 130 and 120 mmHg may be related to an increased likelihood of harmfully low DBP, which can affect the cardiac risk. In other words, care should be taken when considering intensive SBP control in patients with IS or TIA.

## Data Availability Statement

All datasets generated for this study are included in the article/Supplementary Material.

## Ethics Statement

The studies involving human participants were reviewed and approved by the central institutional review board of Beijing Tiantan Hospital. The patients/participants provided their written informed consent to participate in this study.

## Author Contributions

YW contributed to the conception and design of the study. ZC contributed to manuscript drafting and critical revisions of the manuscript. JM, JX, LD, AC, GY, XM, and HL contributed to the acquisition and analysis of data and AW contributed to statistical analysis.

## Conflict of Interest

The authors declare that the research was conducted in the absence of any commercial or financial relationships that could be construed as a potential conflict of interest.

## Supplementary Material

Supplemental Figure 1 Event rates of composite events and stroke recurrence according to DBP categories (from 4 months to 1 year). DBP, diastolic blood pressure.

Supplemental Figure 2 Event rates of composite events and stroke recurrence according to low DBP under different SBP levels (from 4 months to 1 year). SBP, systolic blood pressure; DBP, diastolic blood pressure.
