# Supplementary figures and images for: Effect of Low Diastolic Blood Pressure to Cardiovascular Risk in Patients With Ischemic Stroke or Transient Ischemic Attacks Under Different Systolic Blood Pressure Levels
Source: Front Neurol. 2020 May 27;11:356. doi: 10.3389/fneur.2020.00356 (PMC7267217; doi:10.3389/fneur.2020.00356)

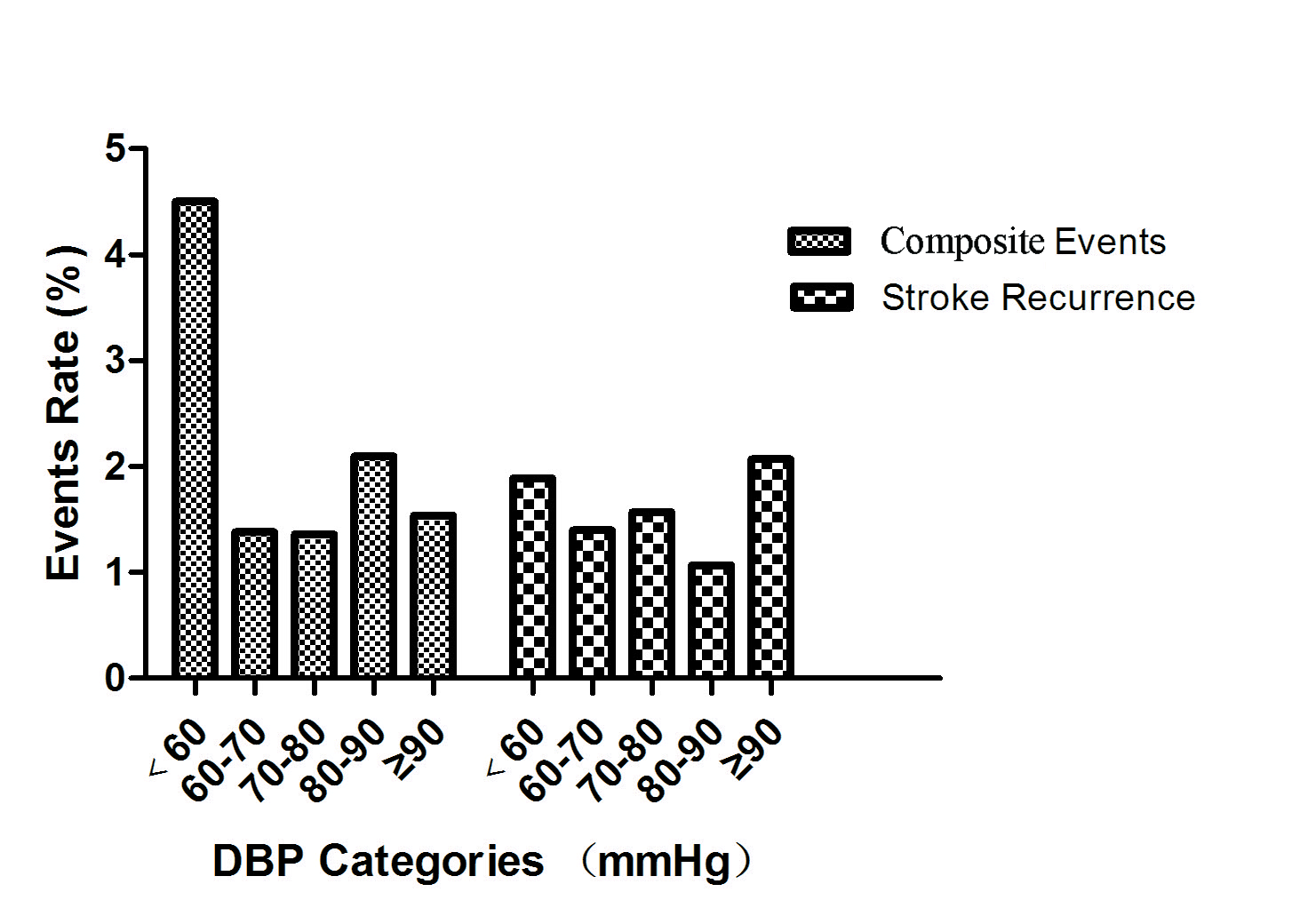

Supplement: Supplemental Figure 1 — Event rates of composite events and stroke recurrence according to DBP categories (from 4 months to 1 year). DBP, diastolic blood pressure. [file Image_1.TIF]

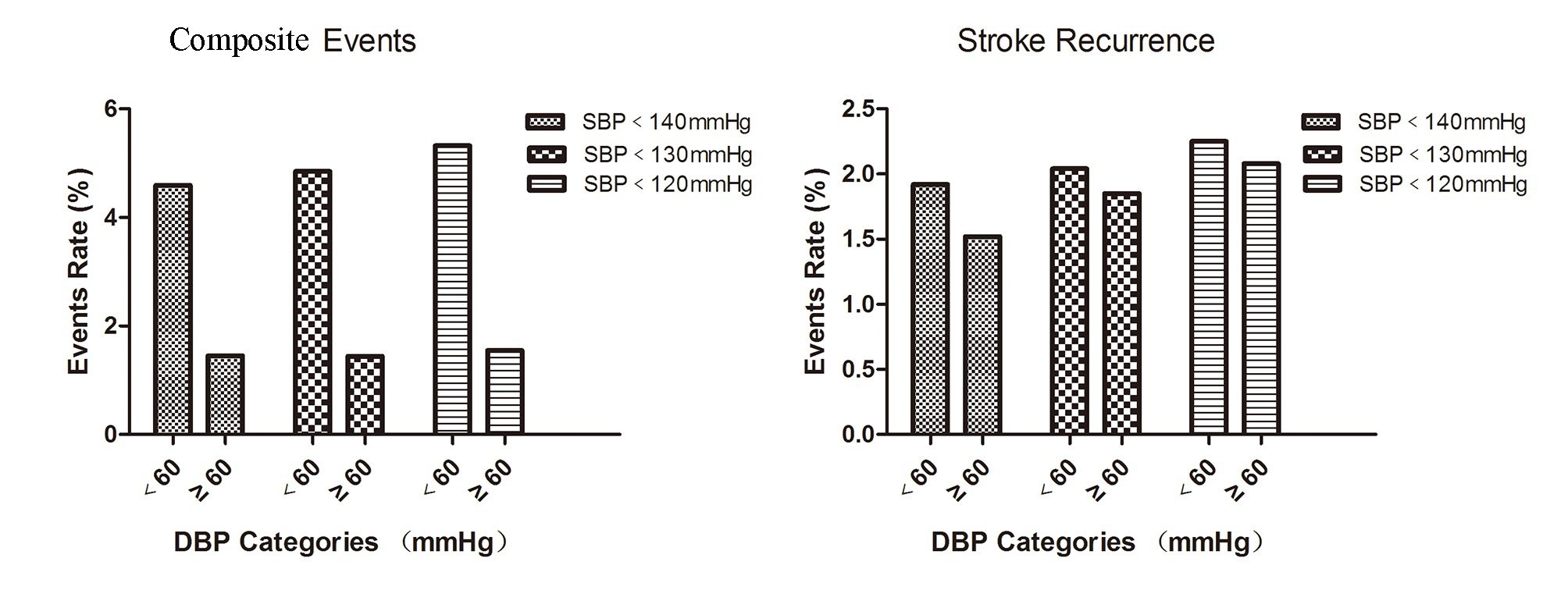

Supplement: Supplemental Figure 2 — Event rates of composite events and stroke recurrence according to low DBP under different SBP levels (from 4 months to 1 year). SBP, systolic blood pressure; DBP, diastolic blood pressure. [file Image_2.TIF]
